# Estimation of Lockdowns’ Impact on Well-Being in Selected Countries: An Application of Novel Bayesian Methods and Google Search Queries Data

**DOI:** 10.3390/ijerph20010421

**Published:** 2022-12-27

**Authors:** Krzysztof Drachal, Daniel González Cortés

**Affiliations:** 1 Faculty of Economic Sciences, University of Warsaw, 00-241 Warszawa, Poland; 2 NEOMA Business School, 75013 Paris, France

**Keywords:** Bayes theorem, COVID-19, mental health, psychological well-being, SARS-CoV-2, quarantine

## Abstract

Lockdowns introduced in connection with the COVID-19 pandemic have had a significant impact on societies from an economic, psychological, and health perspective. This paper presents estimations of their impact on well-being, understood both from the perspective of mental health and considering economic security and similar factors. This is not an easy task because well-being is influenced by numerous factors and the changes happen dynamically. Moreover, there are some obstacles when using the control group. However, other studies show that in certain cases it is possible to approximate selected phenomena with Google search queries data. Secondly, the econometric issues related to the suitable modeling of such a problem can be solved, for example, by using Bayesian methods. In particular, herein the recently gaining in popularity Bayesian structural time series and Bayesian dynamic mixture models are used. Indeed, these methods have not been used in social sciences extensively. However, in the fields where they have been used, they have been very efficient. Especially, they are useful when short time series are analyzed and when there are many variables that potentially have a significant explanatory impact on the response variable. Finally, 15 culturally different and geographically widely scattered countries are analyzed (i.e., Belgium, Brazil, Canada, Chile, Colombia, Denmark, France, Germany, Italy, Japan, Mexico, the Netherlands, Spain, Sweden, and the United Kingdom). Little evidence of any substantial changes in the Internet search intensity on terms connected with negative aspects of well-being and mental health issues is found. For example, in Mexico, some evidence of a decrease in well-being after lockdown was found. However, in Italy, there was weak evidence of an increase in well-being. Nevertheless, the Bayesian structural time series method has been found to fit the data most accurately. Indeed, it was found to be a superior method for causal analysis over the commonly used difference-in-differences method or Bayesian dynamic mixture models.

## 1. Introduction

Well-being is an important concept in various disciplines. However, there is no single definition of this term. Roughly speaking, well-being can be understood as a positive life attitude and feeling good. It is also an interdisciplinary concept with different aspects of well-being studied across a variety of disciplines. For example, physical well-being, economic well-being, social well-being, psychological well-being, etc. Moreover, those different forms of well-being can be closely interlinked [1,2,3]. Events related to the COVID-19 pandemic have shown how important, but difficult to analyze, this category is [4,5,6,7].

Indeed, the first confirmed infections of humans were detected in Wuhan, China in December 2019 and by that time the infection was spreading by the human-to-human channel. In the early stages of the pandemic, the number of cases was doubling almost every 7 days. In January 2019, the virus spread over other regions of China and by the end of the month, the World Health Organization globally warned about it. The same month, the virus came to Europe. The first major restrictions began in China, then, other Asian countries followed. As soon as by the end of April 2020, numerous people in the world were under some form of a lockdown. Indeed, the COVID-19 pandemic resulted in the largest number of simultaneous lockdowns around the world. However, few countries did not use lockdown strategies to reduce the number of infection cases [8]. The virus is believed to have killed between 12 and 22 million people by the middle of 2022, depending on estimations. Moreover, hundreds of millions of people suffer from long COVID [9,10]. The International Monetary Fund estimates that the pandemic will cost the global economy 12.5 trillion USD through 2024 [11]. Moreover, the World Bank estimates that the pandemic has increased the number of people living in extreme poverty to between 119 and 124 million, which is the largest increase since 1990. Additionally, the pandemic increased food insecurity, negatively impacted access to education and social services and had a devastating impact on the labor market and income [12]. 

This paper employs Google search queries in order to estimate the impact of lockdowns on the broadly understood individual well-being of citizens of various countries. Individual well-being is measured at the aggregated level, i.e., by the Internet search queries of certain words in given countries (contrary to individual survey studies). In particular, in the case of well-being, emphasis is placed on mental health issues. However, certain factors which may be linked with social or economic well-being are considered explanatory variables in the employed models. Additionally, the obtained outcomes for different countries are compared.

When the COVID-19 lockdowns were implemented, a clear concern about the mental health, emotional well-being, etc. of citizens emerged [4]. Most of the up-to-date literature indicates numerous negative effects of the introduced lockdowns in the form of psychological issues, stress, an increase in the feeling of being lost, an increase in aggressive thoughts and behaviors, etc. [13]. These feelings are associated with social isolation, fear of being infected, boredom, frustration, lack of access to information, financial loss, links with limitations of physical activity, nutrition patterns, etc. [14,15,16,17,18,19,20,21,22]. Some studies also pointed to long-term effects [7]. Indeed, even before the COVID-19 pandemic, Ref. [23] considered a weighted averaging constructed index based on some specific word search queries addressed to Google Search in analyzing well-being in the United States. In conclusion, they indicated that data from Internet search engines can be a significant supplement to traditional survey research. 

With regard to the COVID-19 pandemic, for example, Ref. [24] also tried to find out how lockdowns affect well-being. However, their analysis was based solely on conventional econometric methods and only covered a narrow selection of European countries. The keywords (i.e., search terms) were selected on the basis of the General Health Questionnaire. Moreover, they made some simple rescaling of the initial Google Trends indices in order to compare them over different time periods (i.e., before and after the pandemic). Unfortunately, the selected countries were hardly diversified. Moreover, as explained later in this paper, the conventional econometric methods can have certain drawbacks in the case of this particular research problem, compared with Bayesian methods, which can serve as better alternatives and offer more efficient tools in this regard. 

This paper tries to answer the question of whether the implemented lockdowns impacted the well-being of various societies in a significant way. In particular, well-being is narrowed down to mental health issues. The addressed changes are proxied by Internet search queries. Secondly, the modeling schemes include several macroeconomic (and other) variables in order to capture the impact on well-being from other sources such as economic conditions, etc. Another advantage of this study is that it analyzes numerous individual countries separately based on a consistent methodology, thereby differentiating this study from certain others concerning this topic. Finally, instead of the more conventionally used difference-in-differences method for causal analysis, this study heavily explores Bayesian methods, especially those able to deal with explanatory variable uncertainty and time-varying parameters. In particular, Bayesian structural time series models and Bayesian dynamic mixture models are employed. 

The implementation of quarantine and lockdown restrictions certainly leads to the disruption of social networks, impacting negatively on the mental health of the general population [25,26,27]. Several studies reported significant negative consequences [28,29,30]. The impact of the pandemic on the mental health of citizens was studied in a variety of ways, mostly reporting huge negative effects; however, adaptations between waves of the pandemic were also observed [31]. Some authors argue that the negative impact on well-being was due to the pandemic itself, and not a result of the lockdowns per se [32,33,34]. In this study, contrary to the difference-in-differences method [35,36] commonly used in this type of research, novel Bayesian methods are used. In particular, the innovative applications of Bayesian structural time series and Bayesian dynamic mixture models are implemented [37,38].

Nevertheless, despite the many advantages, Bayesian methods are characterized by high computational complexity and the necessity to approximate distributions [39]. Fortunately, in the case of the particular methods mentioned herein, the approximations are only at the numerical level of the parameters—not the functional forms of the distributions themselves [38], which is an important advantage in itself, and the computation complexity is at a very acceptable level [37].

The usefulness of information about Internet search queries provided by Google Trends in economics and finance was noted by [40]. They initiated numerous subsequent papers on the application of this kind of information, for example, in forecasting unemployment, real-estate prices, consumer decisions, etc. [41]. For instance, the modeling of the unemployment rate is a very illustrative example of the advantage of Google Trends data and its usefulness in forecasting tasks compared to traditional approaches. Indeed, the data published by statistical offices are available only with a certain significant delay because the necessary information must be collected and properly processed in the traditional way. Meanwhile, the activity of Internet users—what they are searching for—may be an indicator of, for example, the situation that they are actively looking for a job or losing their current employment and are likely to report to the labor office in the very near future. 

Google Trends publishes such data on a continuous basis; therefore, this information is already hidden in their data—before it is reported to, processed, and published by the official statistical bureau. This kind of approach to forecasting has resulted in the so-called nowcasting [41,42,43]. When investigating issues related to the COVID-19 pandemic, such methods have also found application in many aspects of scientific research, including infection predictions [44]. For example, Ref. [45] analyzed the index constructed on the basis of Twitter posts in relation to COVID-19 lockdowns and happiness, using the difference-in-differences method. They found that lockdowns resulted in a decline in happiness, regardless of the type and duration of lockdown, furthermore, severe lockdowns resulted in a greater happiness decline. Ref. [46] analyzed Internet searches in Latin American countries, concluding that searches for insomnia peaked but then declined after a few weeks of lockdown, whereas searches for stress, anxiety, and sadness increased and stayed high. However, they found no change in searches for depression or suicide after the lockdown. 

Indeed, the activity of Internet users may be an indicator that is available much faster than, for example, a traditional survey [47]. It also works well for groups that are difficult to analyze in a traditional way [41,43]. For example, Refs. [23,48] applied averaging procedures to Google Trends indices and showed the usefulness of such data in analyzing well-being in the United States. They showed that data from Internet search engines can be a significant supplement to traditional survey research. However, it should be emphasized that in the case of forecasting the number of infections, models based solely on data from Internet search engines can be unreliable in certain cases. For instance, they can drastically overestimate the number of cases in some periods [49]. When forecasting new cases, the Bayesian structural time series happened to be a very useful tool [50], together with the general use of the Bayesian approach in machine learning techniques [51]. 

The literature mentioned above and related to measuring the impact of lockdowns primarily used data on Internet search queries from the Google search engine. A significant problem, in this case, was the choice of keywords. For example, Ref. [52] argued that the use of Google Trends in such a context is heavily limited, being inconsistent with the controlled traditional survey performed at the same period of time. However, they analyzed only a limited number of keywords. Ref. [53] also questioned the efficiency of using Google Trends to monitor the mental distress of citizens; however, they concluded that these indices can be a proxy for coping and resilience factors. Again, the key factor for the interpretation of the results was the proper selection of Internet search terms. Secondly, their study was limited to only one country. Nevertheless, the general use of unconventional tools in this regard, having in mind the increasing role of the Internet and the use of social media, has become more and more common among researchers [54,55]. 

In order to clarify, the Google Trends index for a given word (i.e., search term) returns an index ranging from 0 to 100. This normalized index expresses the share of the Internet search queries for a given word amongst all the queries addressed to the Google search engine [40]. As a result, using this index requires some subtle treatment. For instance, it can be due to the proper identification of outliers. Indeed, certain inquiries may arise at selected periods of time as a result of political debates or events not directly related to the pandemic. Secondly, there also exists a problem with the language used in searching as some countries have more than one useable language. Moreover, it can happen that certain topics are not searched in a mother tongue language [56]. 

In addition, Google Trends generates an index that measures the search intensity of a given keyword, and it can also generate an index for a whole given topic. However, the topic identification by Google’s algorithms can sometimes be misleading [57]. In any case, the time series for topics and keywords are not always similar. A certain solution to this problem was suggested by [57,58]. Their work, however, concerned the modeling of economic activity and trade. For this purpose, they used the Shapley value from game theory and applied it in machine learning.

Most studies into the effects of the introduced lockdowns used the difference-in-differences method [59], a method in which initially similar populations are observed, which, for external reasons, have been subjected to a different influence of a certain variable. Thus, the equivalents of the control and the experimental groups emerge, which are compared with each other using the regression method. The downside of this method, however, is the need to correctly identify confounding variables. Moreover, there are certain problems with causal relationships, omitted variables, regression toward the mean, or endogeneity [60].

In this context, for example, the level of stress related to welfare may depend on the overall economic condition of a country, its social and public health policies, social security, etc. [61]. Indeed, well-being can be also linked to economic conditions [62,63]. Ref. [64] reviewed how various economic determinants can impact crucial factors affecting the health of citizens. Indeed, there exists evidence that macroeconomic conditions can relate to citizens’ happiness and well-being [65]. However, these relationships can be different for short and long-term periods [66]. Therefore, dynamic Bayesian models are indeed suitable. For example, Ref. [67] analyzed how unemployment (especially in the long term) affects the psychological well-being of citizens of European countries. Similarly, Ref. [68] considered unemployment and GDP as important determinants of individual happiness. Ref. [69] additionally considered inflation in their modeling of subjective well-being, taking into consideration both non-linear and asymmetric effects. Of course, there are still other factors influencing well-being, but it is not possible to reasonably measure them against the relatively short and changing periods of the time horizon of the research proposed here [70,71]. For example, [72], as well as [73], provided a review of various macroeconomic variables on well-being.

The aspects mentioned above require considering certain additional and important variables in the constructed econometric models. Otherwise, the aforementioned problem with omitted variables can occur. It should be noted that, contrary to the common misconception, more explanatory variables do not automatically result in more accurate forecasts [74]. In this regard, there are various methods for variable (feature) selection, as this is a truly subtle issue, such as LASSO regression, Principal Component Analysis, etc. [75]. However, they are less suitable to simultaneously deal with the calculation of the effect of an intervention (i.e., lockdown) on an outcome (i.e., well-being) if used in a direct and simple way. 

Ref. [76] proposed extending the difference-in-differences method with Bayesian inference. Their method takes into account the selection of variables using the spike and slab method [77], in order to estimate which potentially important variables should be included in the model. In addition, the Bayesian approach is used for the estimation of regression coefficients by using standard inference based on prior and posterior distributions and Monte Carlo sampling with Markov chains [78]. This procedure happened to be an efficient method if the number of potential variables is relatively large in relation to the number of observations (time series’ lengths). Moreover, this method leads to unbiased and efficient estimators. As a result, it reveals the great usefulness of incorporating Bayesian feature improvements. However, this method is based on Bayesian hierarchical models, as they were claimed by the authors to be a natural way to account for the very specific structure of the sample that they used and the practical problem that they focused on. In particular, certain observations inside the sample were characterized by correlations due to similar geographic, economical, and demographical conditions. For the problem posed in the currently reported research, such issues are actually not so important. The model constructed by [76] focused strongly on slightly different aspects than those required in the research reported herein.

Within this context, it is also worth mentioning the recently proposed approximate Bayesian inference approach using variational methods from [79,80], which can tackle conjugate exponential family models with latent variables and non-stationarity. This novel scheme is based on hierarchical priors and explicitly models temporal changes in the model parameters. It is able to deal with time-varying parameters and its implementation has important computational advantages. 

As an alternative, Ref. [81] recently proposed the ArCo method (artificial counterfactual approach). This is an interesting method that can be used to assess the causality in the absence of a control group. It is a two-step method in which the first step is based on the selection of variables using the LASSO regression method. However, LASSO regression suffers some problems itself, for instance, when the number of predictors is more than the number of observations. Moreover, if there is a group of variables among which the pairwise correlations are very high, then LASSO tends to arbitrarily select only one variable from the group. Bayesian methods have also been developed recently in this context, and the outcomes seem promising. Therefore, it would be interesting to look at this stage of the analysis—the selection of variables, for instance—on the basis of Bayesian dynamic mixture models [38].

These methods are based on mixture models that take into account both the fact that the model itself (a set of variables describing the phenomenon under study) changes over time and the model’s coefficients (for instance, regression parameters). Moreover, mixture models take into account combining forecasts from numerous component models, instead of selecting just one model. This also turns out to be a useful feature [38,82]. It should be emphasized that one of the alternatives (when studying causal inference) to the difference-in-differences method is the Bayesian structural time series approach. This method combines the Kalman filter, spike and slab, and Bayesian combination of forecasts [37,83]. As a result, these two novel methods would, therefore, be interesting to apply to the current research problem of lockdown impacts on well-being. This is so because, contrary to the difference-in-differences method, for example, they allow the capture of time evolution, full Bayesian inference, and a flexible approach towards various sources of variability such as local trends, seasonality, time-varying parameters, etc., which, as already mentioned, are important features in the current research problem.

In particular, the current paper describes a coherent and uniform approach to the indicated economic and social problem, based on the latest econometric and statistical tools (only just beginning to be used in economic and social sciences). These tools are most appropriate for overcoming the specified problems and nuances related to the modeled phenomenon and seem to be more efficient research tools than the methods used so far. In particular, an attempt is made to address the following problems and questions: how lockdowns affected the mental well-being of citizens; to what extent Google search query information can be used for this purpose; construction of a novel econometric model that combines such a complex problem is proposed, potentially numerous variables, nuances, and limitations related to information from the Google search engine are efficiently tackled; and Bayesian structural time series models and Bayesian dynamic mixture models are checked to see if they are more useful in this regard than conventional methods.

## 2. Data

Due to the data availability, 15 countries were chosen for the analysis. Although some of the data, for instance, those purely connected with COVID-19 cases [84,85,86,87] are available at high frequencies, macroeconomic data are at most at monthly frequencies. Moreover, not every country reports unemployment at monthly frequencies for long enough periods back in time to make the samples used herein consistent with each other. Another limitation comes from uncertainty indices. In fact, analysis solely based on Google search queries and COVID-19 cases could be made for a larger sample; however, the purpose of this study is to also cover also, at least initially in the first steps, country-specific macroeconomic data and uncertainty indices. Although lockdowns are measured in days, using monthly data to estimate their impacts—if required by the availability of other data—does not seem to be a problem [88,89]. The above requirements on data resulted in the possibility to derive adequate time series samples for Belgium (BEL), Brazil (BRA), Canada (CAN), Chile (CHL), Colombia (COL), Denmark (DNK), France (FRA), Germany (DEU), Italy (ITA), Japan (JPN), Mexico (MEX), the Netherlands (NLD), Spain (ESP), Sweden (SWE), and the United Kingdom (GBR). 

These countries are characterized by a high share of Internet users in the total population [90] with an exemption only for Columbia and Mexico. Secondly, Google searches constitute almost all Internet search queries in these countries [91]. The only slight discrepancy is present in Japan. The share of Internet users is more responsible for the possibility of making conclusions from this research over the whole countries’ populations. The share of Google engine in total search engines used in the given country seems to have more effect on the potential conclusions. If using Google searches from a country where Google is not the dominant search engine, then a certain approximation about overall Internet users is made, so that the Google users are representative of all the Internet users. 

In any case, the sample considered herein seems to be a good one. The data quality is high, i.e., it comes from reliable sources and has been continuously used and reviewed by numerous research groups. Moreover, the above analysis shows that the data are indeed representative for the purpose of the research. Secondly, the selected countries represent different geographical regions and cultural dimensions and were responding somehow differently to the COVID-19 pandemic. As a result, the proposed analysis can, indeed, bring a deeper insight into the problem posed in this research. 

Google Trends data [92] were downloaded with the help of the “gtrendsR” R package [93]. Subsequent to previous studies [4,23,24] the following search queries were considered: “afraid”, “apathetic”, “boredom”, “contentment”, “depression”, “divorce”, “fear”, “frustration”, “impairment”, “irritability”, “loneliness”, “nervous”, “panic”, “pissed”, “sadness”, “scared”, “sleep”, “stress”, “suicide”, “tension”, “well-being”, “worry”, and “worthless”. They were translated into a given country’s dominant language. For Belgium, the ordinary average from Dutch and French searches was taken following the method used in the construction of the economic policy uncertainty (EPU) index [94]. For the other countries, the dominant language was selected. This method can at first sight seem slightly inappropriate for Canada and Spain, in which a second language is used by significant minorities [94,95,96]. However, EPU indices for these countries were constructed on the basis of the dominant language only [97,98]. As a result, herein the same rule was followed. 

Short-term interest rates (i_rate) were obtained from [99,100]. In general, they are understood as rates corresponding to short-term government papers on the market and a 3-month horizon. In the case of Chile, some observations were missing, and so, each missing value was replaced with the most recent non-missing value prior to it. Inflation rates (cpi) were obtained from [100], taken as consumer price indices (CPI), representing annual growth rates of prices of a basket of goods and services typically purchased by specific household groups. Unemployment rates (une) were taken from [99,100,101,102]. They are understood as the seasonally adjusted share of unemployed people relative to the total labor force (i.e., the total number of unemployed people plus those in employment). The unemployed are understood as people of working age who are without work, are available for work, and have taken specific steps to find work. Stock prices (stocks) were taken from [100]. They are understood as common shares traded on national or foreign stock exchanges. As the data are monthly, they are averages of daily closing prices, represented by an index set to 100 in the year 2015. 

The geopolitical risk (GPR) was measured by the index of [103]. This index aims to capture acts of violence and competition over territories between states, a wide range of adverse geopolitical events, threats, realizations, and escalations. In broad terms, it captures acts of war, terrorism, international crises, and tensions between states. The index is based on automated text searches of newspapers and is available on a country level. In particular, the Recent Index (as the authors provide various versions of their original index), beginning in 1985, was taken for each country (gpr). 

In addition, the economic policy uncertainty (EPU) indices mentioned already were considered. They are similar to GPR indices but focus on policy-related economic uncertainty [95,97,98,104,105,106,107,108,109]. In the case of Denmark, the extended version of the index was taken as it is claimed by the authors to better capture country-specific issues for Denmark. Unfortunately, this index ends in June 2021.

Global real economic activity (ec_act) was approximated by the Kilian index [110,111,112]. This index is based on business cycles and expresses derivations from the trend. It is based on dollar bulk dry cargo shipping rates, which seems to be a crucial index for measuring activities in global industrial commodity markets. It is a better proxy than real GDP; moreover, it is published monthly [113], contrary to other less frequent traditional measures. 

As this research is focused on the period of the COVID-19 pandemic, following, for instance, Ref. [114] advice, the Brent oil price was taken as a proxy of the global spot oil price (oil). The data were taken from [115]. The Oil spot price was considered because it impacts many economic factors. It is crucial for inflation movements and its price impacts transportation and production costs. Secondly, energy itself is an important factor impacting the well-being of societies [116,117]. 

The time horizon of lockdowns is a subtle issue because of how the term “lockdown” should be defined and understood in a precise manner for the purpose of econometric modeling. Indeed, the problem is to identify the time frames of the introduced lockdowns, understood as periods of quarantine preventives resulting in significant disruption of social networks. They are linked to stay-at-home orders and closing public places, schools, workplaces, etc. While the dates of their implementation are relatively unambiguous, the dates of their ending may be blurry, as their endings were generally spread over time by gradually loosening the restrictions. Yet another problem was the regionalization of restrictions in individual countries. In order to deal with these issues, the dates of lockdowns were taken from [85] in the following way; first of all, the national-level jurisdiction of government restrictions was considered. Secondly, for Brazil, Canada, and the United Kingdom, targeted restrictions were counted, whereas for the other countries they had to be general ones. Finally, for the purpose of this research, the lockdown was understood as the occurrence of any of the following two cases: firstly, an internal movement restriction being in place, and secondly, both schools and workplaces closing at least for some levels or categories of schools or sectors or categories of workers and restrictions on gatherings of 10 people or less. Such a definition is also in line with qualitative discussion and results for no lockdown in Japan and Sweden being detected [118]. 

Regarding COVID-19 cases data [84,85,86,87], they were aggregated from a daily data level to a monthly level. The number of new cases per million citizens (new_cases_per_million) and the number of new deaths per million citizens (new_deaths_per_million) were summed for a given month. Reproduction rate (reproduction_rate) and (pandemic restrictions) stringency index (stringency_index) were averaged over days in a given month. Of course, cases were taken relative to the number of citizens, as the absolute values are not meaningless for the purpose of this research. The ranges of values are reported in Table 1.

For data clearing procedures, for instance, to ascribe 0 values for COVID-19 data-related time series, March 2020 was taken as a breakpoint. Considering data availability and in order to have the same length of the analyzed period for each country, finally, the period between January 2004 and June 2021 was taken as the whole sample. The time series inserted into the models were in monthly frequencies.

## 3. Methodology

It was possible to start the sample in January 2004. This might seem like quite a long look into the past, especially compared to other similar research [4,23]. However, it does not result in any drawbacks (except for potential data availability issues, which actually had not appeared in this case). On the methods applied in the current research, a longer pre-intervention of time series lets the model adequately learn the evolution of time series, i.e., “catch the signal” [119], and its relationship with covariates. 

### 3.1. Bayesian Structural Time Series

Bayesian structural time series models were estimated with the help of the “bsts” R package [83,120]. For the trend component in Bayesian structural time series models, initially, a local linear trend was selected because it assumes that both the mean and the slope of the trend follow random walks; it can better reflect the behavior of the analyzed data. However, other trend specifications were also considered for robustness issues. In order to capture data periodicity, a 12-month seasonal component was added. 

The formal framework of the Bayesian structural time series model, as used in this research (i.e., “local level model”, because, in general, the structure can be more complicated), is as follows: Let $y_{t}$ be the modeled time series (response variable), i.e., Google Trends index for a given word. Then, the observation equation is given as $y_{t}=\mu_{t}+\gamma_{t}+\beta_{t}^{T}x_{t}+\varepsilon_{t}$ with the transition equation $\mu_{t}=\mu_{t-1}+\delta_{t-1}+u_{t}$ and $\delta_{t}=\delta_{t-1}+v_{t}$ and $\gamma_{t}=-\sum_{i=1}^{S-1}\gamma_{t-i}+w_{t}$. The term $\mu_{t}$ represents the trend and captures the time series tendency to move in a particular direction with time. The term $\gamma_{t}$ represents the seasonal component and, herein, represents potential monthly effects. The term $\beta_{t}^{T}x_{t}$ represents the regression and $x_{t}$ is a vector of covariates (the predictors described in the Data section). The error terms $\varepsilon_{t}$, $u_{t}$, and $v_{t}$ are Gaussian and independent. The term $\delta_{t}$ can be understood as the slope of the local linear trend. 

It can be seen that the modeled time series is assumed to be a combination of trend, seasonality, and impact components. The details are described, for example, in papers by [37,83], and the above formulas constitute a state space model. Indeed, the Bayesian structural time series model specification is the one used by [83]. The models were fitted by the Markov chains Monte Carlo algorithms with 1000 iterations. Additionally, a spike and slab method was used on a set of predictors. This method is different from, for instance, LASSO methods. The inclusion probability of each predictor is updated on the basis of Bayesian inference and is used to control for keeping most of the regression coefficients equal to 0 and to prevent spurious regression [83,121]. 

The above models were the first step toward the estimation of causal inference with the help of the “CausalImpact” R package [37]. Indeed, this package performs causal inference through counterfactual predictions using Bayesian structural time series models. The causal impact is understood as the difference between the observed values of the response variable and the unobserved value that would have been observed under no intervention (i.e., lockdown). The unobserved potential values are predicted by Bayesian structural time series models. 

The important drawback of the Bayesian structural time series approach to causal inference is that the control time series themselves must be assumed to not be affected by the intervention. Secondly, it is assumed that the relationship between covariates and treated time series, as established during the pre-intervention period, remains stable. It cannot be, herein, reasonably assumed that the mentioned covariates were not themselves affected by the intervention. Therefore, a two-step procedure was followed; first, Bayesian structural time series models with no covariates were estimated for each of the variables mentioned in the Data section. Secondly, those for which a 95% credible interval of the causal effect included 0, meaning that the causal effect of lockdown (positive or negative) cannot be considered statistically significant, were used as covariates in the next set of models. Nevertheless, it was a priori assumed that ec_act and oil are not impacted, as lockdown decisions were at the country level, but these variables represent the global quantities. Additionally, the stringency_index (because of its construction) was a priori excluded as a possible covariate. In this next step, the testing was based on similar verification of the existence of the causal effect (positive or negative), and, if detected, then Bayesian one-sided tail-area probabilities were reported (which correspond to the one-sided test, checking for the significance of the positive effect). 

For robustness of results, additionally, local level, semi-local linear, and Student local linear trends were considered. The local level trend is the random walk. The semi-local linear trend assumes that the level component behaves like the random walk, but the slope behaves as the AR(1) process. As the stationary AR(1) process is less variable than the random walk, this specification can be better for long-term forecasting. The Student local linear trend assumes that both the level and the slope follow random walks. It is based on uniform priors, contrary to the normal priors for local linear trend specification [37]. 

### 3.2. Difference-in-Differences

Following, for example, [59,122,123,124,125], the difference-in-differences analysis was done with the help of the following equation: $y_{t}=\alpha_{0}+\alpha_{1}\delta_{t}+\alpha_{2}\gamma_{t}+\alpha_{3}\lambda_{t}+\alpha_{4}^{T}x_{t}+\varepsilon_{t}$ with $\delta_{t}$ being a dummy variable representing yearly seasonal effects and $\gamma_{t}$ being a dummy representing monthly seasonal effects. $\lambda_{t}$ is a binary dummy variable equal to 1 in periods during and after the lockdown, and equal to 0 before the lockdown. $x_{t}$ is a vector of covariates as before (but now all possible variables are considered), and $\varepsilon_{t}$ is the error term. The coefficient of an interest is $\alpha_{3}$, as it measures the change in average Internet searches before and after the lockdown. Contrary to the cited research with the panel approach, herein, distinct equations were estimated separately for each country. However, for robustness checks $\delta_{t}$ and $\gamma_{t}$ were used both as dummy variables and as fixed effects factors. Moreover, estimations such as those in the above-cited papers—after merging the dataset into the panel and with fixed country effects—were also performed. In this case, no intervention was assumed in Japan and Sweden. (In all other models, in Japan and Sweden, April 2020 was artificially selected as the contrived intervention period, so the outcomes can serve as robustness checks.) 

In the panel approach, the Google Trends time series were rescaled, following [59], in order to provide time series consistent between different countries. In particular, the original Google Trends time series for a given country and a given search term were divided by their averages from the whole analyzed period. 

Despite the possibility of now considering all variables mentioned in the Data sections as possible covariates, the difference-in-differences method still has other drawbacks. For instance, this method is conventionally restricted to the static regression estimation, not including temporal evolutions. Secondly, it can lead to misleading conclusions if serially correlated data are used. Moreover, this method considers only two change points: pre and post-intervention. It does not analyze how the effect of an intervention evolves with time. Finally, the relationship between covariates and response variables is placed under relatively complicated assumptions [37]. 

Therefore, for the robustness of the results, a few specifications for this method were considered. In particular, the vector $x_{t}$ was considered for contemporaneous impact but also models with the first lags, i.e., $x_{t}$ replaced with $x_{t-1}\;$were considered. Secondly, models with no vector of covariates at all were considered as well as the vector $x_{t}$ itself being considered in a few versions. Of course, the full possible specification (i.e., with all variables listed in the Data section) was also considered. Additionally, *x* = [new_deaths_per_million] and *x* = [new_cases_per_million, new_deaths_per_million, epu, gpr] were also considered. 

### 3.3. Bayesian Dynamic Mixture Models 

Bayesian dynamic mixture models were estimated with the help of the “dynmix” R package [124]. In particular, the mixture estimation with state-space components was considered [38] in order to be more consistent with a Bayesian structural time series approach. The causal inference was estimated mimicking the patterns used in the Bayesian structural time series models estimations. The difference between the observed values of the response variable and the unobserved values that would have been observed under no intervention was considered, whereas the unobserved potential values were predicted with Bayesian dynamic mixture models.

A detailed explanation of Bayesian dynamic mixture models can be found in the original papers by [38] and [82]. Briefly, herein, there are n=12 possible covariates to be considered, i.e., $x=\left[1,x_{1},\ldots ,\;x_{n}\right]\;=\;$[const. term, i_rate, cpi, une, stocks, gpr, epu, ec_act, oil, new_cases_per_million, new_deaths_per_million, reproduction_rate, stringency_index]. As a result, $K=2^{n}$ multilinear regression models (including with the constant term only) can be created by including or not including each of these covariates. In other words, each of the slots in x (except the constant term) can be replaced by 0 or kept as it is above. For clarity, let us name each of these models by an individual component model. 

Each of them can be described as the state-space model. Let k={1,…,K} index them. The k-th state model is represented by the probability density function (pdf) $f_{k}\left(\theta_{t}^{k}|\theta_{t-1}^{k},\delta_{t}^{k}\right)\;$and the output model by the $g_{k}(y_{t}|x_{t}^{k},\varepsilon_{t}^{k})$ pdf, with the response variable $y_{t}=x_{t}^{k}\theta_{t}^{k}+\varepsilon_{t}^{k}$, and t being the time index, $x_{t}^{k}$ is the vector of covariates in the k-th component model, and $\theta_{t}^{k}$ are regression coefficients and $\varepsilon_{t}^{k}$ is the normal noise. $\delta_{t}^{k}$ is assumed to follow the multivariate normal distribution with mean 0 and the specified covariance $W_{t}^{k}$. The recursive estimations are done with the Kalman filter. These component models constitute the mixture model [38]. 

The algorithm of mixture estimation begins with the initial values of regression coefficients equal to 0 and ascribing to each of the components an equal weight 1/K. Then, as the new data are upcoming, the regression coefficients and mixture components weights are re-estimated. This is done according to the Bayesian inference schemes [38]. Due to certain computational issues, the time series were standardized before inserting into the models. Afterward, the predicted values were transformed back to have magnitudes consistent with the original time series and those generated by other models. Means and standard deviations for standardizations were computed on the basis of pre-intervention periods. 

### 3.4. Benchmark Models

Finally, the auto ARIMA models of [126] were taken as the benchmark models to evaluate forecasting accuracies. Additionally, the no-change (naïve) forecasts were also taken. In the case of the auto ARIMA, the number of lags for AR and MA processes are automatically increased in order to generate more accurate fitting to the data. This is evaluated on the basis of the Akaike Information criterion. At the same time, the number of these lags is somehow limited to guarantee stopping of the algorithm.

All the estimated models are listed in Table 2. 

### 3.5. Comparision of the Outcomes

The accuracy of how the estimated models fit the observed data (i.e., residuals) was evaluated with the Root Mean Square Error (RMSE) measure. This is a very common measure, and greatly penalizes higher errors as well as symmetrically penalizing negative and positive errors. Moreover, the quadratic loss function is consistent with the Diebold-Mariano test (DM) and Model Confidence Set procedure (MCS). 

The computations were done with the help of the “MCS” R package [127]. A total of 1000 bootstrapped samples were used to construct the statistic test. The “TR” statistic for the MCS procedure was chosen and a 0.90 confidence level was assumed. The Diebold–Mariano test is used to compare two forecast accuracies. In other words, it checks whether the difference between two forecasts is statistically significant. The test is based on the quadratic loss function. The null hypothesis of the test is that the difference between two forecasts is not significant, whereas the alternative hypothesis is that the selected forecast is more accurate than the second one [128]. In particular, small sample correction by [129] was implemented for this test. 

The MCS procedure is similar to the Diebold–Mariano test, but it is designed to evaluate multiple forecasts [130]. The equal predictive ability of the given set of forecasts is tested. If the null hypothesis that all forecasts have the same accuracy can be accepted, then the procedure stops. Otherwise, a selected forecast is excluded, and the updated set of forecasts is tested again. The whole procedure is recursively repeated. For this procedure the quadratic loss function was also used, and, as a result, all the mentioned methods were based on the same loss function, thereby making the different methods consistent with each other. 

The computational part of this research was done in R [131].

## 4. Results

The analysis was performed for 23 words and 15 countries. As a result, 345 time series were treated as response variables and 345 different models with different modeling methods were estimated. Consequently, it is impossible to report every particular model in detail herein. However, such detailed outcomes can be provided by the author for interested parties. For the sake of clarity, most of the outcomes presented in this section apply to certain averaging; over particular words, over countries, or over models in general (jointly over both countries and words). Nevertheless, individual models, although not reported in detail, were still examined during the research process.

Table 3 reports average values of RMSEs over various words for a given country and a given model. Table 4 reports average values of RMSEs over various countries for a given word and a given model. It can be seen that it is always the BSTS_3 or BSTS_3_l model which minimizes RMSE values out of all the considered models. Indeed, considering separately all 345 time series (for a given word and a given country), in 37% of cases BSTS_3 model, and in 47% of cases BSTS_3_l model, minimized RMSEs out of all the considered models. In the remaining 16% of cases, it was either the BSTS_1, BSTS_1_l, BSTS_2, BSTS_2_l, BSTS_4, or BSTS_4_l model. As a result, it was always some form of BSTS model which minimized RMSE. In particular, whether the “best” fitting model was the one with lagged or not lagged covariates is a minor issue.

More interesting is the fact that, first of all, Bayesian structural time series models happened to beat all other models in terms of fitting the data. Secondly, the semi-local linear trend specification happened to be the “best” one in the case of fitting the data. Actually, as mentioned before [37] this specification can be the better choice in the case of long-term forecasting (and, indeed, the post-intervention period for which the forecasting needed to be done in this research was quite a long period). The semi-local linear specification treats the level component as the random walk, and the slope as the AR(1) process. The stationary AR(1) process is less variable than the random walk. This kind of specification seems to also naturally fit the Internet search query characteristics.

According to these outcomes, the BSTS_3_l (i.e., Bayesian structural time series model with lagged covariates and semi-local linear trend) can be assumed as the “best” fitting model and can be selected as the major model for further analysis. In any case, except when comparing RMSE values, the Diebold–Mariano test outcomes are also reported in Table 5. This table reports the average *p*-values of DM tests over various words for a given country and a given model. The null hypothesis of the test is that the forecasts from the BSTS_3_l model and from the competing model have the same accuracy. The alternative hypothesis is that the forecast from the BSTS_3_l model has greater accuracy. 

It can be seen that the selected model “beats” all other models almost always, except for “beating” the BSTS_3 model. This confirms that whether covariates are lagged or not is a minor issue. In only a few cases, the selected model generated forecasts not significantly more accurate than those from other BSTS-type models, nevertheless, this still confirms that BSTS-type models are superior to other techniques. Similar conclusions can be derived from Table 6, which reports average *p*-values of the DM tests over forecasts for various countries for a given country and a given model compared with the forecast from the corresponding BSTS_3_l model. In addition, the overall analysis of all individual forecast comparisons (for each word and each country separately) confirms that in the overwhelming majority of cases, forecasts from the BSTS_3_l model were significantly more accurate than those from the competing model. 

Finally, the MCS procedure confirms the above conclusions. The testing procedure was performed with the parameters specified in the Methodology section. Table 7 reports the frequency of the presence of a given model in the superior model set created by the MCS procedure. In other words, the MCS procedure was performed for each individual case: a particular word and a particular country. The reported frequencies present how often the given model remained in the superior set of models created for each such individual case. Indeed, the outcomes are not surprising, having in mind the previously stated results of the DM tests. First of all, in the majority of cases, the BSTS_3 model with non-lagged covariates or with lagged covariates remained. The third most frequently remaining model was, however, the NAÏVE model. Nevertheless, it happened only in approximately 15% of cases. All other models remained in less than 10% of cases. Interestingly, the NAÏVE model remained in many more cases than the ARIMA model. Although the BDMM models remained in very few cases, they still remained more often than the DiD models.

Ultimately, despite considering several specifications and versions of DiD models, none of them happened to fit the data in a satisfactory way for a reasonable number of cases. This can be explained by the previously mentioned drawbacks of this method, i.e., that it approaches time-varying regression parameters, fluctuations of the response variable, seasonality, trends, etc., poorly. The BSTS and BDMM methods have an internal model structure incorporation that deals so nicely with various sources of volatility as well as time-varying relationships between response variables and covariates. 

Having in mind the above results, the answers to the initial research problems can be found. For the words “contentment”, “divorce”, “frustration”, “impairment”, “irritability”, “loneliness”, “nervous”, “pissed”, “sadness”, “scared”, “suicide”, and “worthless”, none of the models BSTS_3_l or BSTS_3 detected any causal effect from a lockdown. As a result, the causal effect was present only for approximately 48% of the analyzed words. Secondly, no causal effects for any word were found in BEL, CAN, DNK, JPN, and ESP. Thirdly, the outcomes from the BSTS_3 and BSTS_3_l models are not consistent with each other. Fortunately, neither are they contradictory (i.e., opposite conclusions on causal effects cannot be derived from these two separate models). However, one of the models for certain words and countries detected some causal effect, whereas, for the same country-word combination, the second model did not in numerous cases. The only case that leads to the same conclusions based on both models is a very strong increase in “well-being” and a moderate increase in “stress” searches in Mexico.

Another interesting outcome is that countries with no explicit lockdowns such as Japan and Sweden do not seem to differ from the other 13 analyzed countries. Another important outcome is that the considered models not only detected positive causal effects but also negative ones—meaning that lockdown resulted in a decrease in searches for a given word. The BSTS_3_l models for Italy especially detected such cases (i.e., fewer searches for the words: “afraid”, “boredom”, “depression”, “fear”, “sleep”, ”well-being”, and “worry”). Indeed, BSTS_3_l models for Mexico suggested a decrease in well-being after lockdown, whereas the same model for Italy suggested an increase in well-being (if measured by the aforementioned word searches). In the case of 16 significant causal effects detected by the BSTS_3 model, 4 of them are negative. Except for Brazil, these 4 cases happened for quite developed and safe countries.

As a result, it could be said that it is not a lockdown per se that can decrease well-being, but rather the effect depends on the overall situation in a country: its policies and abilities to provide citizens with safety and protection, whether there is social trust for the government and officials, etc. Finally, the constructed models included covariates, suggesting that the causal effect of a lockdown could have been indirect (for example, it impacted the covariates) rather than direct (i.e., directly impacting word search term indices). Secondly, the model with non-lagged covariates detected more causal effects and more positive causal effects than the model with lagged covariates, which detected less significant causal effects, most of which were negative. Moreover, lagging covariates can be understood as leading to the erasing of causal effects detection, meaning that, overall, the effects, if present, are very immediate and short-term. Herein, monthly data were used, therefore, lagging variables can play a significant role. The exact numerical values are reported in Table 8.

Deriving any conclusions from DiD or BDMM models seems to be flawed because these models generally poorly modeled the data. Despite containing relatively many parameters, they seem to fit the data less than even for the NAÏVE method. 

Finally, the negative effects seen in DEU for “panic” are consistent, for example, with a similar study by [24]. However, the positive effects for “sleep” and “worry” are contradictory to their results. The sign (i.e., positive or negative) of the effect for “well-being” depending on the particular country, found herein, is similar to the outcomes of [24]. Concerning the classification of words, as in [24], the current research weakly suggests that in certain countries a lockdown resulted in increased anxiety issues. At the same time, no significant evidence for social dysfunction and happiness changes was found. 

## 5. Conclusions

The methods employed in this research found little evidence of substantial changes in the Internet search intensity on terms connected with negative aspects of well-being and mental health issues. The Bayesian structural time series method fit the data much better than the difference-in-differences method or Bayesian dynamic mixture models. Moreover, the differences between various specifications of Bayesian structural time series models did not appear to be very important. In any case, the model with lagged explanatory variables and semi-local linear trend specification turned out to be the “best” one. The current research considered various covariates which can also impact well-being. The aim of this was to carefully consider the impact of a lockdown on well-being, both as a direct impact and separating, for example, the impact through the selected covariates (related to economic or policy uncertainty, etc.). The performed study cannot confirm that a lockdown leads to a significant increase in certain word searches in most of the analyzed countries. However, in Mexico, such an effect was indeed found. In Italy, surprisingly, a negative effect was found. This can suggest that it is not a lockdown per se that influenced well-being but rather the overall social background and government actions. For example, in different societies, it can be perceived as a negative restriction of an individual’s freedom, whereas in another country or society, as a way of the government protecting the people. Secondly, the current research was based on monthly data, therefore, strongly focused on long-term effects. Analysis based on more frequent data could suggest different results over shorter periods, but then macroeconomic variables would be an issue. The current research is also vulnerable to lockdown definitions and measures; for example, policies in Belgium or Netherlands are much more unified and consistent across the whole country than those in Brazil or Canada, where different administrative units may have more diversified policies around the country. Within this context, further studies can measure lockdown not only as certain legal policies but also as obeying these laws (which varied in different countries and societies). Moreover, the data used in this research measured certain factors across whole countries (at the aggregated level), so it can be that different regions (for example, big cities vs. rural areas) or age groups were impacted differently as well as certain data can have different quantitative measures when considered over smaller administrative units. Additionally, Google Trends data do not differentiate Internet users; different social or age groups can react differently to lockdowns. In addition, the current study did not find significant differences between countries with explicit lockdowns and those without (such as Sweden). Anyways, this study tackled this research problem in a novel way, i.e., with Bayesian structural time series method. The obtained results are partially consistent with, for example, some previous studies—based on a different methodology—by [24]. However, some outcomes obtained herein are still in a contradiction to their findings. Finally, another interesting continuation of this research would be to analyze not just various search terms as words, but whole topics in Google Trends.

## Figures and Tables

**Table 1:** Ranges of values of COVID-19 data.

Country	New_Cases_per_Million			New_Deaths_per_Million		
	Mean	Min	Max	Mean	Min	Max
BEL	5487.40	0.09	26,716.65	127.89	0.00	592.23
BRA	5110.13	0.01	10,305.46	142.52	0.00	385.02
CAN	2071.95	0.11	6301.08	38.89	0.00	123.99
CHL	4763.79	0.10	10,550.86	96.41	0.00	241.20
COL	5170.33	17.68	16,278.40	129.89	0.31	346.63
DNK	2991.90	0.52	14,277.26	25.70	0.00	142.26
FRA	5143.02	0.08	14,423.42	91.87	0.00	308.84
DEU	2468.63	0.06	7936.40	60.18	0.00	284.55
ITA	3920.49	0.03	15,275.18	117.43	0.00	307.83
JPN	352.41	0.10	1229.59	6.52	0.00	22.34
MEX	1074.44	0.00	3363.72	96.38	0.00	251.26
NLD	5783.48	0.58	16,189.92	60.97	0.00	219.47
ESP	4899.79	0.96	17,431.82	102.70	0.00	343.97
SWE	6310.64	1.38	19,118.80	86.16	0.00	281.89
GBR	3919.81	0.03	19,485.08	104.44	0.00	478.99

**Table 2:** Abbreviations of the estimated models.

Abbreviation	Full Name
BDMM	Bayesian dynamic mixture model (with all possible covariates)
BDMM_l	BDMM model but with lagged covariates
BSTS_1	Bayesian structural time series model with local linear trend
BSTS_2	Bayesian structural time series model with local-level trend
BSTS_3	Bayesian structural time series model with semi-local linear trend
BSTS_4	Bayesian structural time series model with the Student local linear trend
BSTS_1_l	BSTS_1 model but with lagged covariates
BSTS_2_l	BSTS_2 model but with lagged covariates
BSTS_3_l	BSTS_3 model but with lagged covariates
BSTS_4_l	BSTS_4 model but with lagged covariates
DiD_0	Difference-in-differences model with no covariates
DiD_1	Difference-in-differences model with new_deaths_per_million as covariates
DiD_2	Difference-in-differences model with new_cases_per_million, new_deaths_per_million, epu and gpr as covariates
DiD_3	Difference-in-differences model with all possible covariates
DiD_1_l	DiD_1 model with lagged covariates
DiD_2_l	DiD_2 model with lagged covariates
DiD_3_l	DiD_3 model with lagged covariates
DiD_FE_0	DiD_0 model expanded by monthly and yearly fixed effects
DiD_FE_1	DiD_1 model expanded by monthly and yearly fixed effects
DiD_FE_2	DiD_2 model expanded by monthly and yearly fixed effects
DiD_FE_3	DiD_3 model expanded by monthly and yearly fixed effects
DiD_FE_1_l	DiD_FE_1 model with lagged covariates
DiD_FE_2_l	DiD_FE_2 model with lagged covariates
DiD_FE_3_l	DiD_FE_3 model with lagged covariates
DiD_p_0	Panel DiD_FE_0 model
DiD_p_1	Panel DiD_FE_1 model
DiD_p_2	Panel DiD_FE_2 model
DiD_p_3	Panel DiD_FE_3 model
DiD_p_1_l	DiD_p_1 model with lagged covariates
DiD_p_2_l	DiD_p_2 model with lagged covariates
DiD_p_3_l	DiD_p_3 model with lagged covariates
ARIMA	Auto ARIMA model of [126]
NAIVE	The no-change model

**Table 3:** Average values of RMSE over the words.

	BEL	BRA	CAN	CHL	COL	DNK	FRA	DEU	ITA	JPN	MEX	NLD	ESP	SWE	GBR
BDMM	11.91	11.82	11.40	13.45	13.18	15.77	11.28	11.84	12.41	9.22	10.62	12.86	11.80	15.61	10.68
BDMM_l	11.95	11.81	11.44	13.54	13.57	15.96	11.36	11.65	11.91	9.13	10.60	12.86	11.74	15.15	10.56
BSTS_1	7.24	4.94	4.80	6.01	5.74	8.31	5.34	6.28	5.80	3.45	3.97	6.64	6.35	8.71	4.02
BSTS_2	7.04	4.35	4.77	5.95	6.05	9.73	5.13	6.24	5.54	3.23	4.04	6.96	6.13	8.85	3.11
BSTS_3	4.80	3.10	2.92	4.00	3.36	7.59	3.73	4.92	3.84	2.41	2.52	4.65	4.07	7.32	1.82
BSTS_4	6.91	4.65	4.76	5.05	4.75	9.29	5.03	6.28	5.48	2.52	3.31	6.59	5.38	8.22	4.05
BSTS_1_l	7.26	4.97	4.75	6.27	5.99	8.43	5.19	6.35	5.85	3.27	4.11	6.41	6.36	8.76	4.25
BSTS_2_l	6.98	4.42	4.59	6.10	5.94	9.75	4.90	6.33	5.58	2.99	4.24	6.67	6.11	9.03	3.29
BSTS_3_l	4.84	3.27	2.83	4.26	3.37	7.74	3.50	4.92	4.11	2.33	2.29	4.54	4.13	7.32	1.79
BSTS_4_l	6.86	4.81	4.82	4.76	4.52	9.45	4.94	6.52	5.41	2.79	4.00	6.60	5.49	8.24	4.02
DiD_0	10.19	11.73	11.14	11.52	12.17	12.97	10.22	10.84	10.93	9.62	9.90	11.59	10.86	12.62	10.67
DiD_1	10.19	11.73	11.15	11.51	12.17	12.97	10.23	10.83	10.91	9.62	9.84	11.59	10.85	12.62	10.67
DiD_2	10.02	11.49	10.91	11.37	12.01	12.89	10.10	10.69	10.69	9.31	9.60	11.27	10.55	12.53	10.49
DiD_3	9.38	9.68	9.76	10.88	10.75	12.04	9.11	9.45	9.53	8.09	8.53	10.31	9.50	11.91	8.99
DiD_1_l	10.19	11.73	11.13	11.51	12.17	12.97	10.21	10.83	10.93	9.62	9.84	11.58	10.87	12.62	10.67
DiD_2_l	10.02	11.45	10.92	11.43	12.04	12.87	10.11	10.70	10.78	9.30	9.60	11.25	10.57	12.54	10.45
DiD_3_l	9.40	9.74	9.86	10.87	10.61	12.01	9.28	9.55	9.50	8.14	8.72	10.28	9.58	11.97	9.12
DiD_FE_0	8.23	7.92	7.36	8.98	8.84	10.89	7.33	8.05	8.17	6.35	6.97	8.64	7.90	10.44	6.64
DiD_FE_1	8.23	7.91	7.36	8.97	8.82	10.89	7.33	8.05	8.13	6.35	6.91	8.64	7.88	10.44	6.64
DiD_FE_2	8.17	7.85	7.33	8.91	8.77	10.79	7.27	7.99	8.06	6.29	6.84	8.57	7.78	10.38	6.55
DiD_FE_3	7.96	7.64	7.09	8.67	8.48	10.54	7.05	7.71	7.85	6.08	6.64	8.33	7.55	10.17	6.28
DiD_FE_1_l	8.23	7.91	7.35	8.97	8.83	10.89	7.32	8.05	8.18	6.35	6.91	8.64	7.90	10.44	6.63
DiD_FE_2_l	8.19	7.85	7.30	8.91	8.79	10.80	7.28	8.00	8.10	6.31	6.84	8.58	7.82	10.38	6.51
DiD_FE_3_l	7.96	7.58	7.06	8.53	8.42	10.49	7.06	7.72	7.47	6.10	6.68	8.27	7.54	10.15	6.24
DiD_p_0	10.84	11.82	12.01	12.79	13.04	13.97	11.72	12.87	12.44	13.39	10.88	13.82	11.47	13.48	10.89
DiD_p_1	10.85	11.81	11.99	12.78	13.03	13.94	11.73	12.84	12.48	13.38	10.89	13.79	11.50	13.49	10.90
DiD_p_2	10.86	11.79	11.99	12.78	13.05	13.96	11.69	12.87	12.59	13.41	10.66	13.82	11.52	13.48	10.79
DiD_p_3	10.81	11.46	11.98	12.66	12.85	13.92	11.51	12.81	12.53	13.22	10.47	13.51	11.56	13.52	10.81
DiD_p_1_l	10.85	11.82	11.98	12.78	13.03	13.93	11.73	12.84	12.44	13.39	10.88	13.77	11.47	13.48	10.89
DiD_p_2_l	10.86	11.81	11.98	12.79	13.05	13.98	11.65	12.87	12.50	13.42	10.61	13.80	11.45	13.49	10.85
DiD_p_3_l	10.84	11.49	12.06	12.67	12.88	13.88	11.49	12.84	12.37	13.24	10.40	13.47	11.42	13.51	10.89
ARIMA	9.73	9.22	8.93	10.34	10.21	12.39	9.18	9.59	9.41	7.12	8.02	10.15	9.63	11.95	8.35
NAIVE	13.05	12.36	11.49	13.61	13.31	16.95	11.91	12.64	12.55	8.95	10.48	13.62	12.71	15.96	10.65

**Table 4:** Average values of RMSE over the countries.

	Afraid	Apathetic	Boredom	Contentment	Depression	Divorce	Fear	Frustration	Impairment	Irritability	Loneliness	Nervous	Panic	Pissed	Sadness	Scared	Sleep	Stress	Suicide	Tension	Well-Being	Worry	Worthless
BDMM	12.21	15.64	12.37	13.42	9.94	9.75	11.15	15.84	13.21	17.61	11.31	14.62	11.15	12.05	9.62	12.44	7.26	11.34	10.39	11.16	12.50	12.49	14.43
BDMM_l	12.03	15.51	12.56	13.36	9.88	9.88	11.02	15.92	13.45	17.65	11.02	14.73	10.96	12.35	9.38	12.36	6.77	11.22	10.43	11.17	12.53	12.37	14.41
BSTS_1	5.52	9.73	6.22	7.79	3.53	4.20	3.78	9.56	6.85	9.53	4.87	4.36	3.76	7.23	4.34	6.34	1.84	4.48	4.03	4.78	6.08	7.42	8.10
BSTS_2	5.66	9.72	5.99	7.85	3.55	4.40	3.97	9.84	6.62	9.25	5.08	4.24	3.37	6.82	4.33	6.44	1.82	4.68	4.02	4.59	5.86	7.45	8.07
BSTS_3	4.08	7.79	4.02	6.47	2.08	3.14	2.86	7.03	4.07	6.60	3.09	2.82	2.49	4.17	3.16	4.44	1.44	2.68	3.16	2.58	4.45	5.31	5.67
BSTS_4	4.25	8.69	5.80	7.74	3.35	3.78	3.60	9.48	6.52	9.74	4.46	4.33	2.76	6.90	4.11	6.52	1.87	4.20	2.81	4.32	6.02	6.99	7.90
BSTS_1_l	5.81	9.81	6.26	7.41	3.72	4.59	3.99	9.53	6.83	9.47	5.06	4.39	3.85	7.02	4.29	6.56	1.85	4.61	3.58	4.70	6.03	7.43	8.49
BSTS_2_l	5.60	9.78	6.14	7.86	3.48	4.55	4.16	9.64	6.56	9.51	5.12	4.28	4.25	6.48	4.28	6.48	1.82	4.36	3.32	4.48	5.76	7.34	8.01
BSTS_3_l	4.12	7.63	3.88	6.37	1.95	3.32	2.77	6.87	4.15	6.71	3.46	2.92	2.83	4.54	2.97	4.48	1.27	2.69	2.44	2.78	4.45	5.35	5.97
BSTS_4_l	5.11	9.16	6.18	7.02	3.12	3.85	3.77	9.03	6.58	9.30	4.37	4.35	3.06	7.32	4.17	6.65	1.84	4.32	2.68	4.54	5.96	7.34	7.92
DiD_0	11.29	13.25	11.46	10.53	11.19	9.10	10.78	13.58	11.46	14.58	10.10	12.70	10.56	11.10	10.35	11.71	7.75	11.28	9.52	10.89	10.92	10.35	11.59
DiD_1	11.29	13.25	11.46	10.53	11.19	9.10	10.77	13.58	11.45	14.57	10.11	12.70	10.54	11.10	10.34	11.71	7.74	11.26	9.52	10.88	10.85	10.34	11.60
DiD_2	11.05	13.08	11.21	10.47	10.86	8.88	10.53	13.39	11.30	14.44	9.98	12.53	10.34	10.88	10.16	11.48	7.38	11.08	9.32	10.66	10.71	10.10	11.50
DiD_3	9.90	12.24	9.98	10.08	8.83	7.96	9.50	12.66	10.64	13.58	9.09	11.57	9.23	9.59	8.75	10.19	5.71	9.28	8.11	9.34	10.09	9.50	10.96
DiD_1_l	11.28	13.25	11.44	10.53	11.18	9.10	10.76	13.58	11.45	14.58	10.11	12.71	10.53	11.10	10.35	11.72	7.76	11.26	9.52	10.88	10.87	10.33	11.59
DiD_2_l	11.09	13.01	11.25	10.50	10.87	8.92	10.48	13.41	11.28	14.52	9.97	12.56	10.36	10.88	10.13	11.49	7.45	11.07	9.23	10.68	10.72	10.15	11.47
DiD_3_l	10.04	12.23	10.12	10.11	8.88	8.07	9.46	12.68	10.66	13.63	9.10	11.49	9.36	9.64	8.82	10.18	5.88	9.44	8.08	9.46	10.12	9.52	10.92
DiD_FE_0	8.64	11.26	8.81	9.41	5.85	6.79	7.80	11.16	8.46	12.07	7.48	7.42	8.04	8.59	7.21	8.65	3.90	6.61	6.88	6.38	7.88	8.79	10.05
DiD_FE_1	8.64	11.25	8.81	9.41	5.85	6.79	7.79	11.16	8.45	12.06	7.48	7.42	8.02	8.59	7.20	8.65	3.87	6.59	6.88	6.36	7.81	8.78	10.05
DiD_FE_2	8.54	11.15	8.72	9.36	5.80	6.70	7.71	11.10	8.41	11.95	7.43	7.35	7.95	8.53	7.16	8.57	3.84	6.53	6.76	6.34	7.78	8.67	10.00
DiD_FE_3	8.28	10.86	8.38	9.19	5.62	6.47	7.38	10.89	8.25	11.65	7.23	7.14	7.48	8.29	6.97	8.40	3.61	6.38	6.56	6.14	7.62	8.44	9.77
DiD_FE_1_l	8.65	11.25	8.81	9.41	5.85	6.79	7.80	11.15	8.45	12.06	7.48	7.43	8.01	8.59	7.21	8.65	3.90	6.59	6.88	6.36	7.83	8.79	10.05
DiD_FE_2_l	8.61	11.12	8.76	9.37	5.80	6.74	7.71	11.07	8.40	12.01	7.43	7.36	7.97	8.55	7.15	8.57	3.87	6.56	6.72	6.30	7.79	8.72	9.97
DiD_FE_3_l	8.35	10.84	8.42	9.20	5.53	6.47	7.34	10.78	8.19	11.65	7.17	7.06	7.49	8.24	6.92	8.26	3.59	6.35	6.51	6.01	7.57	8.38	9.53
DiD_p_0	13.86	13.98	12.26	12.64	12.07	10.48	12.67	14.44	13.34	15.73	11.14	15.41	11.17	13.08	10.93	12.84	7.98	13.13	10.03	10.71	12.60	10.94	12.90
DiD_p_1	13.86	13.96	12.27	12.63	12.07	10.48	12.67	14.44	13.34	15.73	11.14	15.41	11.17	13.08	10.93	12.84	7.98	13.11	10.03	10.70	12.64	10.92	12.90
DiD_p_2	13.85	13.91	12.20	12.62	11.98	10.47	12.71	14.39	13.27	15.70	11.09	15.41	11.17	12.80	10.92	12.83	7.98	13.09	9.99	10.67	13.23	10.89	12.90
DiD_p_3	13.61	13.88	11.79	12.61	11.93	10.29	13.11	14.26	13.31	15.71	10.96	15.40	11.02	12.73	10.77	12.71	7.81	12.59	9.55	10.62	13.08	10.89	12.88
DiD_p_1_l	13.86	13.96	12.27	12.62	12.06	10.48	12.67	14.43	13.34	15.73	11.14	15.41	11.17	13.08	10.93	12.84	7.98	13.09	10.03	10.68	12.47	10.94	12.90
DiD_p_2_l	13.83	13.89	12.18	12.67	11.94	10.46	12.91	14.40	13.28	15.70	11.08	15.42	11.18	12.79	10.91	12.85	7.98	13.06	9.95	10.63	12.88	10.92	12.89
DiD_p_3_l	13.63	13.90	11.82	12.64	11.95	10.28	13.27	14.25	13.33	15.74	10.97	15.44	11.02	12.77	10.75	12.76	7.85	12.54	9.53	10.60	12.44	10.93	12.87
ARIMA	9.40	12.73	9.94	10.50	7.75	7.92	9.06	12.71	10.49	13.93	8.63	10.71	8.66	9.68	7.65	9.93	5.02	8.76	7.72	8.74	10.00	10.03	11.17
NAIVE	12.07	16.78	13.16	14.38	10.52	10.68	11.18	17.10	13.97	18.76	11.19	14.19	10.61	13.18	9.41	12.86	6.18	11.61	10.02	12.03	13.20	13.25	15.36

**Table 5:** Average *p*-values of the DM test over the words.

	BEL	BRA	CAN	CHL	COL	DNK	FRA	DEU	ITA	JPN	MEX	NLD	ESP	SWE	GBR
BDMM	0.00	0.00	0.00	0.00	0.00	0.00	0.00	0.00	0.00	0.00	0.00	0.00	0.00	0.00	0.00
BDMM_l	0.00	0.00	0.00	0.00	0.00	0.00	0.00	0.00	0.00	0.00	0.00	0.00	0.00	0.00	0.00
BSTS_1	0.00	0.09	0.00	0.12	0.03	0.18	0.01	0.05	0.03	0.02	0.00	0.00	0.04	0.08	0.09
BSTS_2	0.00	0.09	0.00	0.09	0.04	0.00	0.04	0.05	0.09	0.04	0.04	0.00	0.04	0.08	0.12
BSTS_3	0.66	0.52	0.48	0.44	0.50	0.68	0.36	0.48	0.52	0.31	0.16	0.47	0.48	0.45	0.26
BSTS_4	0.00	0.17	0.02	0.13	0.15	0.11	0.06	0.14	0.21	0.29	0.07	0.00	0.19	0.20	0.04
BSTS_1_l	0.00	0.01	0.00	0.05	0.00	0.10	0.00	0.05	0.00	0.05	0.00	0.00	0.00	0.04	0.00
BSTS_2_l	0.04	0.08	0.00	0.04	0.00	0.00	0.08	0.00	0.05	0.08	0.00	0.00	0.00	0.03	0.02
BSTS_4_l	0.01	0.03	0.00	0.18	0.17	0.07	0.08	0.04	0.17	0.23	0.00	0.00	0.19	0.13	0.05
DiD_0	0.00	0.00	0.00	0.01	0.00	0.00	0.01	0.00	0.01	0.01	0.01	0.00	0.01	0.01	0.00
DiD_1	0.00	0.00	0.00	0.01	0.00	0.00	0.01	0.00	0.01	0.01	0.00	0.00	0.01	0.01	0.00
DiD_2	0.00	0.00	0.00	0.01	0.00	0.00	0.01	0.00	0.01	0.01	0.00	0.00	0.00	0.01	0.00
DiD_3	0.00	0.01	0.00	0.01	0.00	0.00	0.01	0.00	0.01	0.01	0.00	0.00	0.00	0.01	0.01
DiD_1_l	0.00	0.00	0.00	0.01	0.00	0.00	0.01	0.00	0.01	0.01	0.00	0.00	0.01	0.01	0.00
DiD_2_l	0.00	0.00	0.00	0.01	0.00	0.00	0.01	0.00	0.01	0.01	0.00	0.00	0.01	0.01	0.00
DiD_3_l	0.00	0.01	0.00	0.01	0.00	0.00	0.01	0.00	0.00	0.01	0.00	0.00	0.01	0.01	0.00
DiD_FE_0	0.00	0.00	0.00	0.00	0.00	0.00	0.01	0.00	0.01	0.01	0.00	0.00	0.00	0.01	0.00
DiD_FE_1	0.00	0.00	0.00	0.00	0.00	0.00	0.01	0.00	0.01	0.01	0.00	0.00	0.00	0.01	0.00
DiD_FE_2	0.00	0.00	0.00	0.00	0.00	0.00	0.01	0.00	0.01	0.01	0.00	0.00	0.00	0.01	0.00
DiD_FE_3	0.00	0.00	0.00	0.00	0.00	0.00	0.01	0.00	0.01	0.01	0.00	0.00	0.00	0.01	0.00
DiD_FE_1_l	0.00	0.00	0.00	0.00	0.00	0.00	0.01	0.00	0.01	0.01	0.00	0.00	0.00	0.01	0.00
DiD_FE_2_l	0.00	0.00	0.00	0.00	0.00	0.00	0.01	0.00	0.01	0.01	0.00	0.00	0.00	0.01	0.00
DiD_FE_3_l	0.00	0.00	0.00	0.00	0.00	0.00	0.01	0.00	0.00	0.01	0.00	0.00	0.00	0.01	0.00
DiD_p_0	0.00	0.00	0.00	0.01	0.00	0.00	0.01	0.00	0.01	0.01	0.00	0.00	0.01	0.01	0.00
DiD_p_1	0.00	0.00	0.00	0.01	0.00	0.00	0.01	0.00	0.01	0.01	0.00	0.00	0.01	0.01	0.00
DiD_p_2	0.00	0.00	0.00	0.01	0.00	0.00	0.01	0.00	0.01	0.01	0.00	0.00	0.01	0.01	0.00
DiD_p_3	0.00	0.01	0.00	0.01	0.00	0.00	0.01	0.00	0.01	0.01	0.00	0.00	0.01	0.01	0.00
DiD_p_1_l	0.00	0.00	0.00	0.01	0.00	0.00	0.01	0.00	0.01	0.01	0.00	0.00	0.01	0.01	0.00
DiD_p_2_l	0.00	0.00	0.00	0.01	0.00	0.00	0.01	0.00	0.01	0.01	0.00	0.00	0.01	0.01	0.00
DiD_p_3_l	0.00	0.01	0.00	0.01	0.00	0.00	0.01	0.00	0.01	0.01	0.00	0.00	0.01	0.01	0.00
ARIMA	0.00	0.01	0.00	0.01	0.00	0.00	0.01	0.00	0.01	0.01	0.00	0.01	0.01	0.01	0.01
NAIVE	0.00	0.00	0.00	0.00	0.00	0.00	0.00	0.00	0.01	0.01	0.00	0.00	0.00	0.00	0.01

**Table 6:** Average *p*-values of the DM test over the countries.

	Afraid	Apathetic	Boredom	Contentment	Depression	Divorce	Fear	Frustration	Impairment	Irritability	Loneliness	Nervous	Panic	Pissed	Sadness	Scared	Sleep	Stress	Suicide	Tension	Well-Being	Worry	Worthless
BDMM	0.00	0.00	0.00	0.00	0.00	0.00	0.00	0.00	0.00	0.00	0.00	0.00	0.00	0.00	0.00	0.00	0.00	0.00	0.01	0.00	0.00	0.00	0.00
BDMM_l	0.00	0.00	0.00	0.00	0.00	0.00	0.00	0.00	0.00	0.00	0.00	0.00	0.00	0.00	0.00	0.00	0.00	0.00	0.01	0.00	0.00	0.00	0.00
BSTS_1	0.08	0.00	0.00	0.08	0.11	0.16	0.07	0.00	0.00	0.00	0.07	0.07	0.26	0.01	0.00	0.00	0.00	0.00	0.07	0.00	0.02	0.00	0.11
BSTS_2	0.08	0.00	0.00	0.07	0.07	0.19	0.13	0.00	0.00	0.00	0.01	0.00	0.26	0.01	0.00	0.00	0.00	0.00	0.07	0.07	0.00	0.04	0.13
BSTS_3	0.48	0.32	0.38	0.39	0.49	0.57	0.52	0.50	0.53	0.45	0.59	0.43	0.74	0.63	0.30	0.36	0.08	0.51	0.26	0.67	0.49	0.37	0.33
BSTS_4	0.26	0.18	0.07	0.07	0.05	0.37	0.20	0.00	0.00	0.00	0.24	0.01	0.37	0.06	0.08	0.00	0.03	0.06	0.20	0.19	0.04	0.13	0.12
BSTS_1_l	0.03	0.00	0.00	0.09	0.00	0.07	0.00	0.00	0.00	0.00	0.01	0.01	0.14	0.06	0.06	0.00	0.00	0.00	0.01	0.00	0.02	0.00	0.00
BSTS_2_l	0.03	0.00	0.00	0.07	0.00	0.12	0.00	0.00	0.00	0.00	0.01	0.00	0.16	0.06	0.00	0.00	0.00	0.00	0.00	0.07	0.07	0.00	0.07
BSTS_4_l	0.20	0.07	0.00	0.13	0.05	0.30	0.14	0.07	0.00	0.06	0.25	0.01	0.27	0.01	0.09	0.00	0.04	0.00	0.21	0.07	0.02	0.00	0.07
DiD_0	0.01	0.00	0.01	0.02	0.00	0.00	0.00	0.00	0.00	0.00	0.00	0.00	0.01	0.01	0.00	0.00	0.00	0.00	0.02	0.00	0.01	0.00	0.01
DiD_1	0.01	0.00	0.01	0.02	0.00	0.00	0.00	0.00	0.00	0.00	0.00	0.00	0.01	0.01	0.00	0.00	0.00	0.00	0.02	0.00	0.00	0.00	0.01
DiD_2	0.01	0.00	0.01	0.02	0.00	0.00	0.00	0.00	0.00	0.00	0.00	0.00	0.01	0.01	0.00	0.00	0.00	0.00	0.02	0.00	0.00	0.00	0.01
DiD_3	0.01	0.00	0.01	0.02	0.00	0.00	0.00	0.00	0.00	0.00	0.01	0.00	0.01	0.01	0.00	0.00	0.00	0.00	0.03	0.00	0.00	0.00	0.01
DiD_1_l	0.01	0.00	0.01	0.02	0.00	0.00	0.00	0.00	0.00	0.00	0.00	0.00	0.01	0.01	0.00	0.00	0.00	0.00	0.02	0.00	0.00	0.00	0.01
DiD_2_l	0.01	0.00	0.01	0.02	0.00	0.00	0.00	0.00	0.00	0.00	0.00	0.00	0.01	0.01	0.00	0.00	0.00	0.00	0.02	0.00	0.00	0.00	0.01
DiD_3_l	0.01	0.00	0.01	0.02	0.00	0.00	0.00	0.00	0.00	0.00	0.01	0.00	0.01	0.01	0.00	0.00	0.00	0.00	0.02	0.00	0.00	0.00	0.00
DiD_FE_0	0.01	0.00	0.01	0.01	0.00	0.00	0.00	0.00	0.00	0.00	0.00	0.00	0.01	0.01	0.00	0.00	0.00	0.00	0.02	0.00	0.00	0.00	0.01
DiD_FE_1	0.01	0.00	0.01	0.01	0.00	0.00	0.00	0.00	0.00	0.00	0.00	0.00	0.01	0.01	0.00	0.00	0.00	0.00	0.02	0.00	0.00	0.00	0.01
DiD_FE_2	0.00	0.00	0.00	0.01	0.00	0.00	0.00	0.00	0.00	0.00	0.00	0.00	0.01	0.01	0.00	0.00	0.00	0.00	0.02	0.00	0.00	0.00	0.01
DiD_FE_3	0.00	0.00	0.00	0.01	0.00	0.00	0.00	0.00	0.00	0.00	0.00	0.00	0.01	0.01	0.00	0.00	0.00	0.00	0.02	0.00	0.00	0.00	0.01
DiD_FE_1_l	0.01	0.00	0.01	0.01	0.00	0.00	0.00	0.00	0.00	0.00	0.00	0.00	0.01	0.01	0.00	0.00	0.00	0.00	0.02	0.00	0.00	0.00	0.01
DiD_FE_2_l	0.00	0.00	0.01	0.01	0.00	0.00	0.00	0.00	0.00	0.00	0.00	0.00	0.01	0.01	0.00	0.00	0.00	0.00	0.02	0.00	0.00	0.00	0.01
DiD_FE_3_l	0.00	0.00	0.01	0.01	0.00	0.00	0.00	0.00	0.00	0.00	0.00	0.00	0.01	0.01	0.00	0.00	0.00	0.00	0.02	0.00	0.00	0.00	0.00
DiD_p_0	0.01	0.00	0.01	0.02	0.00	0.00	0.00	0.00	0.00	0.00	0.00	0.00	0.01	0.01	0.00	0.00	0.00	0.00	0.02	0.00	0.00	0.00	0.01
DiD_p_1	0.01	0.00	0.01	0.02	0.00	0.00	0.00	0.00	0.00	0.00	0.00	0.00	0.01	0.01	0.00	0.00	0.00	0.00	0.02	0.00	0.00	0.00	0.01
DiD_p_2	0.01	0.00	0.01	0.02	0.00	0.00	0.00	0.00	0.00	0.00	0.00	0.00	0.01	0.01	0.00	0.00	0.00	0.00	0.02	0.00	0.00	0.00	0.01
DiD_p_3	0.01	0.00	0.01	0.02	0.00	0.00	0.00	0.00	0.00	0.00	0.00	0.00	0.01	0.01	0.00	0.00	0.00	0.00	0.02	0.00	0.00	0.00	0.01
DiD_p_1_l	0.01	0.00	0.01	0.02	0.00	0.00	0.00	0.00	0.00	0.00	0.00	0.00	0.01	0.01	0.00	0.00	0.00	0.00	0.02	0.00	0.00	0.00	0.01
DiD_p_2_l	0.01	0.00	0.01	0.02	0.00	0.00	0.00	0.00	0.00	0.00	0.00	0.00	0.01	0.01	0.00	0.00	0.00	0.00	0.02	0.00	0.00	0.00	0.01
DiD_p_3_l	0.01	0.00	0.01	0.02	0.00	0.00	0.00	0.00	0.00	0.00	0.00	0.00	0.01	0.01	0.00	0.00	0.00	0.00	0.02	0.00	0.00	0.00	0.01
ARIMA	0.01	0.00	0.01	0.02	0.00	0.00	0.00	0.00	0.00	0.00	0.01	0.00	0.01	0.01	0.01	0.00	0.00	0.00	0.03	0.00	0.00	0.00	0.00
NAIVE	0.00	0.00	0.01	0.01	0.00	0.00	0.00	0.00	0.00	0.00	0.00	0.00	0.01	0.00	0.00	0.00	0.00	0.00	0.01	0.00	0.00	0.00	0.01

**Table 7:** Frequency of the presence of a given model in the superior model set created by MCS procedure.

Model	freq.
BSTS_3_l	68.12%
BSTS_3	64.35%
NAÏVE	15.36%
BSTS_4	9.57%
BSTS_4_l	6.38%
BDMM	4.06%
BDMM_l	3.48%
DiD_p_3	3.48%
DiD_1_l	3.19%
BSTS_1	2.61%
BSTS_2	2.32%
BSTS_1_l	2.03%
BSTS_2_l	2.03%
DiD_FE_1_l	1.74%
DiD_FE_0-	1.45%
DiD_p_2_l	1.45%
DiD_p_3_l	1.45%
ARIMA	1.16%
DiD_0	1.16%
DiD_2	1.16%
DiD_FE_2	1.16%
DiD_FE_2_l	1.16%
DiD_p_2	1.16%
DiD_FE_3_l	0.87%
DiD_p_1_l	0.87%
DiD_1	0.58%
DiD_2_l	0.58%
DiD_FE_1	0.58%
DiD_p_1	0.58%
DiD_3	0.29%
DiD_FE_3	0.29%
DiD_p_0	0.29%

**Table 8:** Relative effects detected by BSTS_3_l and BSTS_3 models (columns representing words with no relative effects found in any country are deleted).

	Afraid	Apathetic	Boredom	Depression	Fear	Panic	Sleep	Stress	Tension	Well-Being	Worry
	no lag										
BRA				−1.71							
CHL								0.86			
COL		2.47					0.19		0.36		
FRA									0.17		
DEU						−0.43	0.12				
ITA											
MEX				0.60			0.21	1.05	0.53	11.22	
NLD											−0.50
SWE											−0.47
GBR									0.17		
	lagged										
BRA											
CHL											
COL											
FRA											
DEU											
ITA	−0.99		−1.00	−1.01	−0.99		−1.01			−1.01	−1.00
MEX								1.25		9.62	
NLD											
SWE											
GBR											

## Data Availability

All data sources are described and cited in the Data section of this paper and publicly available. Due to copyright issues (for instance, public republishing) the particular dataset and source code used for the computations are available from the Corresponding Author upon a request.
